# Temporomandibular disorders and the use of oral splints among elite sport athletes: a questionnaire-based cross-sectional study in Germany

**DOI:** 10.1007/s00784-025-06434-x

**Published:** 2025-07-02

**Authors:** Cordula Leonie Merle, Theresa Antonia Rott, Angelika Rauch, Bernd Wolfarth, Jan Wüstenfeld, Daniel Hellmann, Rainer Haak, Gerhard Schmalz, Dirk Ziebolz

**Affiliations:** 1 Department of Prosthetic Dentistry, UKR University Hospital Regensburg, Franz-Josef-Strauß-Allee 11, 93053 Regensburg, Germany; 2 Dept. of Cariology, Endodontology and Periodontology, University of Leipzig, Leipzig, Germany; 3 Department of Sports Medicine, Humboldt University, Charité University Medicine, Berlin, Germany; 4 Institute of Sports Science, Humboldt University, 10099 Berlin, Germany; 5 Department of Sports Medicine, Institute of Applied Training Science, Leipzig, Germany; 6 Department of Prosthodontics, University of Würzburg, Würzburg, Germany; 7 Department of Conservative Dentistry and Periodontology, Brandenburg Medical School Theodor Fontane (MHB), Geschwister-Scholl-Str. 36, 14776 Brandenburg an der Havel, Brandenburg an der Havel, Germany; 8 Institute of General Medicine, Faculty of Medicine, Leipzig University, Philipp-Rosenthal-Str. 55, 04103 Leipzig, Germany

**Keywords:** Sports, Sports dentistry, Oral health, Aligner, Competitive sports, TMD prevalence

## Abstract

**Objective:**

This retrospective, monocentric, questionnaire-based cross-sectional study investigated self-reported symptoms of temporomandibular disorders (TMDs) and oral splint use among elite German athletes.

**Materials and methods:**

Elite athletes (all sports except disability sports, 18–35 years old) who completed a questionnaire on oral health in sports between May 2020 and April 2021 were included. The assessed parameters included anamnestic TMD symptoms (3Q/TMD), oral splint use (presence, reason, wearing habits), current stress levels (scale 1–10), current training load (hours/week), and oral health-related quality of life (OHRQoL; OHIP-G5). Data from 337 elite athletes (23 ± 4 years, 50% female) were analysed. The prevalence of self-reported TMD symptoms was compared across sexes, orofacial trauma risk levels, and types of sports activity.

**Results:**

10% of the athletes reported TMD symptoms, with a significant sex difference (male: 5%, female: 15%; *p* = 0.006). There was no significant difference based on orofacial trauma risk (*p* = 0.941) or activity type (*p* = 0.913). A higher proportion of athletes with self-reported TMD symptoms reported impaired OHRQoL (OHIP > 0: *p* < 0.001), whereas there were no differences in stress levels and training load. Oral splint use was reported by 26% of the athletes.

**Conclusions:**

10% of elite German athletes require further diagnostic evaluation regarding possible TMD, which might be comparable to the prevalence of TMD among the general population. One quarter of the athletes reported having oral splints. Clinical Relevance: TMD, bruxism, and oral splints should be routinely considered in sports dental care.

## Introduction

Recently, there has been an increase in research on the impact of oral conditions on athletic performance, and various oral health deficits in elite athletes have been reported [1–3]. Oral pain is prevalent among athletes and may impair their performance [4]. One cause of orofacial pain is temporomandibular disorders (TMD) which include musculoskeletal, neuromuscular, and joint diseases. A previous systematic review reported widely varying prevalence rates of TMD in athletes (12–100%) across different studies, with a trend towards a higher prevalence in professional athletes [5]. Since all available data focused on contact sports, the systematic review emphasized a potentially lower prevalence in other sports disciplines [5] because the increased occurrence of orofacial injuries in contact sports could promote the development of TMD [6]. Consequently, additional studies should consider additional disciplines beyond contact sports and perform comparisons between contact and noncontact sports [5]. Furthermore, strength athletes may be at increased risk for TMD. Studies have shown that resistance training athletes frequently report dental clenching during exercise [7, 8], a behaviour classified as awake bruxism [9]. Awake bruxism, in turn, has been associated with an increased likelihood of developing TMD [10]. Given this association, a higher prevalence of TMD in strength athletes is plausible. Regardless of the exact prevalence, TMD is characterized by pain and functional limitations as leading symptoms [11]. In a group of 133 patients, myogenous TMD was characterized by an average pain intensity of 29% on a visual analogue scale and a dration of 5.5 h per day; furthermore, the pain was described as ‘present but partly ignorable’ [12]. Accordingly, TMD patients often experience impaired oral health-related quality of life (OHRQoL) [13, 14]. Given that pain and impaired OHRQoL can affect overall well-being, it is plausible that these factors also impact athletic performance.

Consequently, the prevalence of TMD in athletes, as well as early diagnosis and treatment strategies, warrants further attention in sports medicine. In this context, oral splints are also of interest in sports, as they represent an established therapeutic approach for TMD [15]. Additionally, splints are widely used for managing bruxism and orthodontic treatment (e.g., aligners). Specialized oral splints, known as ‘sports performance splints’, are currently promoted to increase athletic performance. However, the results of studies on their effectiveness remain inconsistent [16].

The present questionnaire-based cross-sectional study aimed to assess the prevalence of self-reported TMD symptoms, their impact on oral health-related quality of life, and the use of oral splints among elite German athletes. It was hypothesized that (1) a higher proportion of athletes in strength sports would report TMD symptoms than athletes in other disciplines (endurance, situational, technical-compositional) and (2) that a higher proportion of athletes in sports with a high risk of orofacial trauma (contact/collision sports) would report TMD symptoms than those engaging in lower-risk sports. With respect to oral splints, it was hypothesized that while a high proportion of athletes have oral splints, only a few regularly wear sports performance splints.

## Methods

This retrospective monocentric questionnaire-based cross-sectional study investigated various aspects of oral health in sports. The questionnaires were part of the annual sports medical examination at the Institute of Applied Training Science (IAT), Leipzig, Germany. The local Ethics Committee of the medical faculty of Leipzig University, Germany, approved the study (No. 075/23-ek). This study was reported in accordance with the strengthening the reporting of cross-sectional studies (STROBE) guidelines [17].

### Participants

All elite athletes from various sports who had attended the IAT for their annual sports medical examination between May 2020 and April 2021 were invited to complete the questionnaire. All the athletes were informed verbally and in writing about the scientific use of their data and provided general written informed consent (independent of this study). Data from elite athletes aged 18 to 35 years of both sexes who had provided written consent and had completed the questionnaire were included. Elite athletes were defined as members of squads or the National League. The exclusion criteria were as follows: missing written consent, age under 18 years or over 35 years, and being a recreational or disability athlete. No further exclusions were applied to represent the full spectrum of elite athletes in Germany. Previous orofacial trauma was recorded and included in the analysis.

### Questionnaire and data collection

The standardized questionnaire covered the following areas: oral health behaviour, oral hygiene, symptoms of periodontitis, symptoms of TMD, oral splints, and orofacial injuries [18]. The questionnaire was developed by an expert group of dentists, sport medicals and athletes. It combined published questionnaires, previously established questions and additional study-specific items. The questionnaire consisted of closed-ended questions, some allowing multiple answers. This manuscript examines the section on TMD symptoms and oral splints.

The symptoms of TMD were assessed according to the previously validated 3Q/TMD screening questions: “Do you have pain in your temple, face, jaw or jaw joint once a week or more?”, “Do you have pain once a week or more when you open your mouth or chew?” and “Does your jaw lock or become stuck once a week or more?” [19]. The participants were asked whether they had an oral splint. Moreover, the reasons for wearing and wearing habits were investigated. Additionally, current stress (rated on a numerical rating scale from 1 to 10) and current training load (hours per week) were assessed. Oral health-related quality of life (OHRQoL) was evaluated using the validated short version of the Oral Health Impact Profile (OHIP-G5) [20]. The OHIP-G5 consists of five questions with five answer options (never, hardly ever, occasionally, fairly often, and very often). This tool assesses four dimensions: orofacial function, orofacial pain, orofacial appearance and psychosocial impact. Higher total scores indicate more severely impaired OHRQoL [21]. Additionally, the extent of oral impact on sports was assessed on a 5-point scale [22]. General characteristics (sex, age, details on sport) were extracted from the sports medicine records. To analyse usability, a pretest with five athletes (mean age: 22.6 years, 3 female) was conducted in April 2020. An expert revised the questionnaire on the basis of observations from the think-aloud strategy. The questionnaire was self-administered in the waiting room.

### Statistical analysis

Descriptive analysis was performed under exclusion of missing data. Statistical analyses were conducted using SPSS for Windows, version 29.0 (IBM Corp., Armonk, U.S.A.). Subgroup analyses were performed to examine the proportion of self-reported TMD symptoms based on sex (male vs. female), risk for orofacial traumata (HT: high, contact/collision sports; MT: medium, limited contact/others; LT: low risk) [23], and sports activity type (endurance, strength, technical-compositional vs. situational sports including ball and combat sports). Additional subgroup analyses were conducted to compare self-reported stress, training load, and impaired OHRQoL (OHIP > 0) between individuals with TMD symptoms (at least one positive response in 3Q/TMD) and those without TMD symptoms. The Kolmogorov‒Smirnov test revealed a non-normal distribution (*p* < 0.05). Therefore, between-group comparisons were performed using the Fisher test, Fisher Freeman Halton test, or Mann‒Whitney U test (two-sided; p value < 0.05). A multivariable binary logistic regression analysis was conducted, including sex, splint use, sports type, elevated risk of orofacial trauma, and reported orofacial trauma.

## Results

### Participating athletes (Table 1)

As part of their routine sports medical care, 465 adult elite athletes were invited to complete the questionnaire. Of a total of 355 completed questionnaires, 18 were excluded for the following reasons: six athletes answered the questionnaire twice (only the first-year response was included), eight athletes exceeded the age limit (> 35 years), four participated in disability sports, one was missing a pseudonym, and one was classified as a recreational athlete. In total, data from 337 athletes were included (50% female, mean age: 23 ± 4 years). The characteristics of the participants are displayed in Table 1. The study included more than 30 different sport disciplines, mainly endurance sports (67%, 16% strength, 10% technical-compositional, 8% situational sports). Most disciplines posed a low risk for orofacial trauma (LT: 80%, MT: 14%, HT: 6%).

**Table 1:** Characteristics of the study participants

Domain		All athletes
**Number of participants ** [n (%)]		337 (100.0)
**Age in years** [mean ± SD]		23.0 ± 3.9
**Sex female** [n (%)]		169 (50.1)
**Activity type** [n (%)]	Endurance	224 (66.5)
	Strength	55 (16.3)
	Technical-compositional	32 (9.5)
	Situational	26 (7.7)
**Sports type** [n (%)]	Individual sports	306 (90.8)
	Team sports	31 (9.2)
**Orofacial trauma risk** [n (%)]	High (Contact/collision sports)	20 (5.9)
	Medium (Limited-contact/others)	48 (14.2)
	Low	269 (79.8)
**Squad** [n (%)]	Olympia squad	38 (11.3)
	Perspective squad	147 (43.6)
	Extension squad	15 (4.5)
	Youth squad 1	88 (26.1)
	Youth squad 2	7 (2.1)
	State squad	28 (8.3)
	Team sport squad	2 (0.6)
	National league	4 (1.2)
	Further	8 (2.4)
**Training history** (years) [mean ± SD]		9.7 ± 4.3
**Training units** (events) **per week** [mean ± SD]		9.6 ± 3.5
**Training time** (hours) **per week** [mean ± SD]		18.7 ± 6.8

**n**: number of participants, **SD**: standard deviation,

### Survey results

*TMD symptoms*: 10% of the athletes responded affirmatively to at least one of the 3Q/TMD screening questions (Table 2). The most common symptom was pain in the temporal or facial region, the jaw or the jaw joint (76%). A total of 45% of the participants reported limitations in mandibular function, and 27% reported pain during mandibular movements. A significant sex difference was observed (male: 5%, female: 15%; *p* = 0.006). No significant differences were identified between different activity types (technical-compositional: 7%, endurance: 9%, strength: 13%, situational: 15%; *p* = 0.913) or orofacial trauma risk levels (HT: 10%, MT: 10%, LT: 10%, *p* = 0.941).

**Table 2:** Self-reported prevalence of symptoms associated with temporomandibular disorder (TMD) according to 3Q/TMD.^16^ Missing data are due to unanswered questions

Domain			*n* (%)
Do you have **pain in your temple**,** face**,** jaw or jaw joint** once a week or more? (*n* = 332)			25 (7.5)
Do you have **pain when you open the mouth or chew** once a week or more? (*n* = 334)			9 (2.7)
Does your **jaw lock or become stuck** once a week or more? (*n* = 334)			15 (4.5)
**At least one symptom** (3Q/TMD positive)	Total number (*n* = 332)		33 (9.9)
	by sex	Women (*n* = 166)	24 (14.5)^†^*
		Men (*n* = 166)	9 (5.4) ^†^*
	by activity type	Endurance (*n* = 221)	20 (9.0)^‡^
		Strength (*n* = 54)	7 (13.0)^‡^
		Technical-compositional (*n* = 31)	2 (6.5)^‡^
		Situational (*n* = 26)	4 (15.4)^‡^
	by orofacial trauma risk	High (Contact/collision sports) (*n* = 20)	2 (10.0)^§^
		Medium (Limited-contact/others) (*n* = 48)	5 (10.4)^§^
		Low (*n* = 264)	26 (9.8)^§^

^†^*p* = 0.006 (Fisher test), ^‡^*p* = 1.000 (Fisher–Freeman–Halton test), ^§^*p* = 0.913 (Fisher–Freeman–Halton test), *significant difference (*p* < 0.05)

*OHRQoL*: 15% of the participants reported an impact on OHRQoL. The median OHIP-5 sum score was 0 (IQR: 0;1). Table 3 shows the distribution of answers. Approximately 13% of the athletes reported oral impacts on sports performance (median: 0; IQR: 0;0).

**Table 3:** Oral health-related quality of life according to the OHIP-5 [21]

Parameter		Rating [*n* (%)]					
Dimension	Question	Never (Rating 0)	Hardly ever (Rating 1)	Occasionally (Rating 2)	Fairly often (Rating 3)	Very Often (Rating 4)	Missing answer
**Oral Functi** **on**	Difficulty chewing	292 (86.6)	35 (10.4)	5 (1.5)	0 (0.0)	0 (0.0)	5 (1.5)
	Less flavour in food	328 (97.3)	5 (1.5)	0 (0.0)	0 (0.0)	0 (0.0)	4 (1.2)
**Orofacial Pain**	Painful aching in your mouth	272 (80.7)	51 (15.1)	8 (2.4)	0 (0.0)	0 (0.0)	6 (1.8)
**Orofacial Appearance**	Uncomfortable about appearance	282 (83.7)	37 (11.0)	11 (3.3)	3 (0.9)	0 (0.0)	4 (1.2)
**Psychosocial Impact**	Difficulty doing usual jobs	331 (98.2)	2 (0.6)	0 (0.0)	0 (0.0)	0 (0.0)	4 (1.2)

### Subgroup analyses

A significantly higher proportion of athletes with TMD symptoms reported limitations in OHRQoL (OHIP > 0 in 59% of *n* = 33; sum score: median: 1, IQR: 0;2, maximum: 4) than did those without TMD symptoms (27% of *n* = 300; *p* < 0.001; sum score: median: 0; IQR: 0;1, maximum: 6). The reported oral impacts on sports performance did not differ significantly between athletes with and without TMD symptoms (24% of *n* = 33, median: 0; IQR 0;0 vs. 12% of *n* = 299; median: 0, IQR 0;0; *p* = 0.078). No significant differences were found in stress levels (5.7 ± 1.8 vs. 5.3 ± 2.3; *p* = 0.309) or weekly training hours (16.4 ± 7.0 vs. 18.9 ± 6.3; *p* = 0.112) between athletes with and without TMD symptoms.

### Logistic regression

Logistic regression revealed that female sex (aOR = 2.65, *p* = 0.021) and the use of oral splints (aOR = 3.76, *p* < 0.001) were significantly associated with TMD symptoms (Table 4). No significant associations were found for sport type or orofacial trauma risk.

**Table 4:** Logistic regression: factors associated with self-reported TMD symptoms

Predictor	Adjusted Odds Ratio	95% CI (approx.)	*p* value
**Sex (female)**	2.65	1.15–6.13	0.021
**Oral splint**	3.76	1.76–8.05	< 0.001
**Elevated orofacial trauma risk**	0.60	0.18–2.00	0.408
**Reported orofacial trauma**	0.75	0.26–2.15	0.561
**Strength sports**	0.42	0.08–2.10	0.284
**Endurance sports**	0.67	0.07–6.56	0.729
**Technical-compositional sports**	0.53	0.09–3.14	0.445
**Situational sports**	0.36	0.10–1.25	0.119

**Table 5:** Ownership and usage of oral splints. Missing data are due to unanswered questions

Domain		*n* (%)
**Presence - Total number** (*n* = 334)		87 (26.0)
**Reason** [n (% of participants having splints)] (*n* = 84)	Bruxism	46 (54.8)
	Orthodontic therapy	30 (35.7)
	Temporomandibular disorders	2 (2.4)
	Performance splint	11 (13.1)
**Frequency of use** [n (% of participants having splints)] (*n* = 87)	Daily	37 (42.5)
	Several times a week	11 (12.6)
	Irregularly	27 (31.0)
	Currently no more	12 (13.8)
**Time of use** [n (% of participants having splints)] (*n* = 83)	While sleeping	72 (86.7)
	The entire day	7 (8.4)
	While sporting	4 (4.8)

*Use of oral splints*: Approximately 25% of the athletes reported having an oral splint. Self-reported reasons and usage patterns are displayed in Table 5. Eleven athletes (3%) reported the ownership of a sports performance splint, with six indicating that they regularly use the splint.

## Discussion

The current study provides an initial overview of self-reported TMD symptoms and oral splints in a large cohort of elite German athletes. 10% of the athletes reported pain or functional limitations typically associated with TMD. Additionally, 26% reported having an oral splint. Only 3% (*n* = 11) had sports performance splints, and only four of all the athletes wore a splint during sports.

By examining responses to the 3Q/TMD, the present study specifically focused on treatment needs [19]. The data on self-reported TMD symptoms indicate that one in ten athletes should undergo a clinical examination and might require treatment for TMD. A recently published survey reported a much higher prevalence of TMD symptoms in female competitive German athletes (52% o *n* = 46 athletes from handball, soccer, volleyball, lifeguarding, open water swimming, swimming, triathlon, judo, Latin dance, rowing) [24]. However, these data cannot be directly compared, as different questions have been applied. Notably, Freiwald et al. (2022) [24] also included temporomandibular joint noises, which had a high prevalence (37%). However these conditions typically do not involve pain and therefore usually do not require treatment [25]. Additionally, they are considered to have low diagnostic reliability. Furthermore, it must be considered that the previous study included only female athletes [24], whereas women in the current study presented a significantly higher prevalence of TMD symptoms (15% vs. 5%; *p* = 0.00). The higher prevalence of TMD in women has been widely discussed [26], particularly in treatment-seeking populations of TMD patients [27] but also in routine dental check-ups, as shown in a Swedish study that used 3Q/TMD screening questions [28]. This sex difference was also confirmed in the present study by multivariable logistic regression, in which female sex remained a significant predictor of self-reported TMD symptoms (Table 4). Both athletes with higher sporting levels (competitive vs. recreational) and those with higher training frequencies reported less frequent TMD symptoms [24]. This finding is consistent with the lower self-reported TMD symptom rate in the highly trained elite athletes in the present study. Moreover, most studies indicate a higher TMD prevalence in elite athletes than in recreational athletes or nonathletes [5], highlighting inconsistencies in the available data. Additionally, clinical screening in a previous study in young athletes revealed a greater prevalence of TMD in competitive athletes (46%) than in recreational athletes (25% *p* = 0.014) [29]. Conversely, in a study of winter endurance athletes, neither clinical (32% vs. 41%, *p* = 0.505) nor anamnestic screning (10% vs. 16%, *p* = 0.541) results were signifcantly dfferent from those of a control group comprising nonathletes [30]. These discrepancies reflect the generally inconsistent findings on TMD in athletes, with reported prevalence rates ranging from 12 to 100% [5]. The comparison of the different studies is challenging because of the different methods used. Thus, future studies should ensure methodological consistency by using standardized evidence-based diagnostic tools, such as the DC/TMD criteria, to improve comparability [5]. However, all the summarized studies focused on contact sports [5]. One hypothesis suggests that orofacial violent impacts in contact sports may contribute to TMD symptoms [6], leading to a higher prevalence of TMD in these athletes [5]. The results of the current trial did not support this hypothesis. This is in line with a more recent study that also found no significant association between orofacial trauma and TMD [31]. Additionally, the hypothesis of higher proportions of self-reported TMD symptoms in athletes in strength sports must be rejected. Furthermore, neither stress nor weekly training hours appear to have influenced the presence of TMD symptoms.

Overall, the data indicate that approximately 10% of elite German athletes experience functional limitations or pain and should undergo further diagnostic and, if necessary, treatment. This result aligns with a meta-analysis on TMD prevalence in the general population, which reported a prevalence of approximately 15% (ranging between 1.5% and 30%) [26]. A limitation of the present study is that no direct comparison with nonathletes was possible. Future research should therefore include a control group of nonathletes to better assess whether extensive athletic activity constitutes a risk factor for TMD.

To the best of the authors’ knowledge, no evidence is available on whether TMD symptoms affect performance at the elite sporting level. However, the present study suggests that athletes’ OHRQoL may be impacted by TMD symptoms. This finding aligns with systematic reviews that described impaired OHRQoL in TMD patients compared with individuals without TMD [13, 14]. This association is plausible given the similarity in questioning: the 3Q/TMD screening asks about pain in the temple, face, jaw, or jaw joint; pain during opening or chewing; and jaw locking, whereas the OHIP-5 assesses painful aching in the mouth (dimension orofacial pain, item 3) and difficulties in chewing (dimension orofacial function, item 1). Since these typical symptoms are characteristic of TMD [11], their impact on the OHRQoL dimensions of oral function and orofacial pain [21] is inherent. In the present cohort, more than 13% of the athlets reported such impacts. Consequently, TMD screening should be integrated into sports dental care. If screening results are positive, a comprehensive clinical functional analysis should follow. Depending on the diagnosis, appropriate treatment may include oral splints. A systematic review revealed that various therapeutic interventions can positively influence OHRQoL in TMD patients [32]. This highlights the potential for improving athletes’ OHRQoL through appropriate treatment following a TMD diagnosis. While direct effects on athletic performance have not been empirically demonstrated, reducing TMD-related discomfort could theoretically provide indirect benefits. In this context, the potential influence of oral splints on performance warrants further investigation beyond the specific debate on performance splints. In contrast, 16% of the athletes reported discomfort with their appearance. (OHIP-G5, item 4). Since this is unrelated to TMD, it may indicate other oral health concerns among the athletes.

To the best of the authors’ knowledge, this study is the first to present data on the use of oral splints, including performance splints, in athletes. Consequently, no comparisons with existing data on athletes can be made. Nevertheless, the reported rate of 26% appears remarkably high. It remains unclear whether this figure is specific to Germany or reflects a more general trend or an international trend in elite athletes. One possible explanation might lie in the high standard of dental care available to athletes in Germany, which has also been reflected in other oral health indicators, such as caries prevalence [30, 33], where German athletes have shown favourable results compared with international data [2]. Previous studies reported that oral splints are the most frequent therapy for TMD and bruxism in Germany [34, 35], and the use of these occlusal appliances is commonly recommended [36]. In the present study, 26% of the athletes reported having an oral splint, which is relatively high compared with the general population data and may reflect the widespread implementation of preventive and therapeutic strategies regarding TMD in German sports dentistry. Moreover, multivariable logistic regression revealed that splint use was significantly associated with self-reported TMD symptoms, suggesting that athletes with symptoms are more likely to receive or use oral splints. However, owing to the cross-sectional nature of this study, no conclusions can be drawn regarding causality or the temporal relationship between symptom onset and splint use. In the present study, only two athletes cited pain as a TMD symptom leading to their splint. Approximately 55% reported bruxism as the reason. This high prevalence may be linked to the idea that, for some individuals, jaw clenching as a form of awake bruxism is part of their physiological repertoire for coping with demanding motor tasks [37]. However, distinguishing between TMD and bruxism as self-reported reasons for their splints may have been difficult. Nevertheless, in view of the high reported proportion of athletes with oral splints (26%), oral splints and bruxism should be considered in sports dentistry. Therefore, regular screening for TMD should be conducted. Athletes with suspected TMD should receive thorough functional diagnostics (Axis I) and psychosocial evaluations (Axis II), e.g., brief DC/TMD (bDC/TMD) [38]. This combination is particularly important for identifying individuals experiencing high psychosocial burden, which may increase the risk of chronicity [39]. If treatment is needed, the key points of INfORM/IADR in TMD management should be considered [40], emphasizing patient-centred care, a biopsychosocial approach, and conservative treatment. On the basis of self-reported data in this cohort, bruxism may be prevalent. In particular, in these cases, initial management should prioritize education and behavioural strategies [41]. Psychological interventions and physiotherapy should be considered [39]. Under no circumstances should a splint be routinely considered the first step towards irreversible occlusal changes [39, 42]. Additionally, depending on the design, oral splints also pose a risk of potential irreversible effects [39]. Care providers should ensure evidence-based education to prevent misuse and unnecessary interventions [39]. In the context of sports, athletes may be particularly vulnerable to overtreatment due to performance-related expectations and misinformation regarding oral appliances. In this study, 10% of the athletes reported using sports performance splints. Given the lack of clear scientific evidence for their efficacy [16], the use of these splints requires critical evaluation. Sports medicine professionals and dentists must ensure evidence-based education to prevent misuse and unnecessary interventions. The promotion of performance splints should be critically assessed within evidence-based sports dentistry.

A particular strength of the current study is the high number of included athletes at the elite level. Furthermore, a large variety of different sports have been included, which is a major benefit of the investigation, since the vast majority of available data focus on contact sports [5]. The present data enable the comparison of different sport types for the first time.

However, the structure of elite German sports and the use of monocentric recruiting at the IAT might have caused selection bias. As part of local cooperation, the IAT was chosen as the study setting because of its role as Germany’s central research institute for elite sports, offering access to a nationwide cohort of high-performance athletes across various disciplines. Nevertheless, some sport types, such as team sports and contact/collision sports, are underrepresented, and the subgroups might be too small to reveal potential associations. This underrepresentation limits the generalizability of our findings, particularly concerning sports with elevated risk for orofacial trauma. This may also explain why no significant associations were found for sport type or trauma risk in the logistic regression analysis. Finally, the use of a survey-based approach must be discussed. TMD is detected less often by anamnesis (self-reported) than by clinical examination [26]. For athletes, this discrepancy between self-perceived symptoms and clinical examination could be even greater because they could have a changed body perception with a lower awareness of such symptoms [30]. Furthermore, the reliance on self-reported data introduces potential biases in general, including self-report bias and recall bias. The self-administered nature of our questionnaire may have further reinforced this effect, as respondents could not clarify uncertainties. This might have further influenced the accuracy of the responses. In the general population, screening using the 3Q/TMD enables the detection of pain-related TMD [19]. As the 3Q/TMD specifically assesses the presence of symptoms at a single point in time, recall bias with respect to the current presence of painful TMD is minimized. However, this methodology may underestimate the overall prevalence of TMD, as symptoms could have occurred at other time points. Given the potential influence of psychosocial distress [39], the recorded prevalence at this single point in time may not fully reflect the true burden of TMD among athletes. Periods of increased psychosocial strain, such as during competitions, could lead to a temporary increase in symptoms that might not be captured in this single assessment. Psychosocial factors play crucial roles in the aetiology and management of TMDs [43, 44], with studies reporting high prevalence rates of anxiety, depression, and somatization among affected individuals [45, 46]. In the present study, these factors were not assessed due to capacity constraints, representing a limitation. Moreover, this absence of standardized psychosocial assessment may have further restricted the ability to fully capture the multifactorial nature of TMD in this cohort. This is particularly relevant, as standardized psychosocial assessments remain underutilized in TMD research. Therefore, future studies should consider repeated assessments over a season combined with validated psychosocial measures to provide a more comprehensive understanding of symptom variation and contributing factors over time. Future research should also aim to address these limitations by incorporating clinical examinations alongside anamnestic questions and psychosocial measures leading to a proper diagnosis, e.g., according to DC/TMD. The inclusion of a comprehensive clinical assessment based on the DC/TMD protocol would allow for a subclassification of TMD (e.g., muscle disorders, disc displacements, arthralgia) [47], which could yield valuable additional insights. However, the present study is unable to offer more specific information because of the exclusive use of the screening questionnaire. Furthermore, a more balanced representation of different sports disciplines should be ensured while also collecting detailed information about the sport [5]. Moreover, the validity of data regarding oral splints must be considered limited. It remains uncertain whether athletes accurately recalled and reported the indications for their oral splint. In particular, the differentiation between splints for bruxism and those for TMD management is challenging for nonexperts, especially since the initial indication may have been made years prior, which was not recorded in the present study. To increase the validity of future studies, incorporating data from athletes’ treating dentists would be beneficial. Longitudinal studies could investigate the effects of TMD therapy on OHRQoL and athletic performance. Furthermore, future studies should investigate these aspects in international cohorts to assess potential cultural or systemic differences in sports dental care.

### Perspective

At least 10% of elite German athletes require further diagnostic evaluation regarding possible TMD. Thus, the prevalence of TMD among elite German athletes appears to be similar to that in the general population within the same age range. A quarter of the interviewed elite athletes reported having an oral splint, primarily due to bruxism or orthodontic therapy. This could indicate a high level of dental care provision. Given these findings, temporomandibular disorders, bruxism, and oral splints should be integrated into sports dental care.

## Data Availability

The data supporting this study’s findings are available from the corresponding author upon reasonable request.
